# Mitochondrial Fe-S cluster biogenesis, frataxin and the modulation of susceptibility to drug-induced cardiomyopathy

**DOI:** 10.18632/aging.100238

**Published:** 2010-11-20

**Authors:** Michael N. Sack

**Affiliations:** NHLBI Center for Molecular Medicine, National Institutes of Health, Bethesda, MD 20892, USA

The role of energy deficits in the development and progression of heart failure is well-established and cardiac high-energy phosphate levels correlate directly with survival in cardiomyopathy patients [1]. In light of these clinical observations it has been posited that the modulation of mitochondrial function may be a feasible strategy to ameliorate heart failure. Aspects of mitochondrial function that have been manipulated to investigate this include: modulation of energy substrate utilization; activation of mitochondrial biogenesis; induction of mitochondrial antioxidant defenses and suppression of mitochondrial death programs including apoptosis and mitochondrial permeability transition. As briefly discussed below, targeting distinct aspects of these programs result in variable levels of success and suggest that our understanding of the role of the mitochondria in the pathophysiology remains incomplete.

Pharmacologic approaches to modulate mitochondrial function have, to date, been limited and have recently been reviewed [2,3]. To identify potential novel therapeutic targets, genetic approaches have been used to directly modulate mitochondrial programming with the subsequent assessment of cardiac effects. These include the genetic augmentation of PGC-1α, the master regulator of mitochondrial biogenesis. Here, somewhat counterintuitively, acute and chronic upregulation of PGC-1α results in deleterious cardiac phenotype [4,5]. In contrast, the overexpression of mitochondrial transcription factor A ameliorates the development of ischemia-induced heart failure [6]. The genetic modulation of substrate utilization similarly exhibit divergent effects. The upregulation of atranscription factor promoting fatty acid oxidation, a known major cardiac source of energy, is detrimental [7], whereas the promotion of glucose uptake enhances cardiac tolerance to ischemia [8]. Other approaches include augmentation of anti-oxidant enzymes, e.g., by the upregulation of cardiac manganese superoxide. These transgenic mice are protected against doxorubicin-induced [9] and diabetic cardiomyopathy [10]. Augmentation of cardiac anti-apoptotic programming by the overexpression of Bcl-2 similarly restores mitochondrial function and reduces cardiomyopathy in desmin null mice [11]. In light of incomplete characterization of proteins involved in the mitochondrial permeability transition (MPT) the genetic modulation of this program to prevent cardiomyopathy has to my knowledge not being explored. However, pharmacologic inhibition of MPT by cyclosporine A attenuates cardiomyopathy and mitochondrial genomic mutagenesis in mice harboring a cardiac specific defect in mitochondrial DNA proofreading [12]. Taken together, these pharmacologic and genetic approaches are beginning to discern distinct levels at which mitochondrial reprogramming may have beneficial effects in preventing the development and progression of heart failure.

An intriguing new finding, by Shultz et al is published in AGING regarding the induction of frataxin. Mutations in frataxin result in the development of Friedreich's Ataxia, an inherited neurodegenerative disease associated with the development of severe cardiomyopathy. Frataxin, itself is involved in mitochondrial iron-sulphur cluster biogenesis which functions, in part, to incorporate appropriate amounts of iron into mitochondrial proteins including aconitase and succinate dehydrogenase [13]. Whether frataxin functions as an iron-chaperone protein or plays a regulatory role in controlling iron and sulphur flux within mitochondria is not yet completely characterized. Nevertheless, the study by Shultz and colleagues [14] shows that increased cardiac frataxin enhances tricarboxylic acid cycle function resulting in increased cardiac ATP, NADH, NADPH and reduced glutathione levels. This array of features is consistent with an enhanced bioenergetic capacity and increased antioxidant defenses. The authors go on to demonstrate that overexpression of frataxin is cardioprotective against doxorubicin-induced cardiomyopathy. This intriguing study shows that the modulation of the mitochondria at the fundamental level of integrating cofactors required for protein functional integrity have beneficial effects in disease processes that are exacerbated by mitochondrial dysfunction. This study further highlights the complexity of mitochondrial function and adds a new level of regulation operational in the pathophysiology of heart failure that may be amenable to therapeutic modulation.
